# Supplementary material for: Validation of a Brief Screening Instrument for Chemical Intolerance in a Large U.S. National Sample
Source: Int J Environ Res Public Health. 2021 Aug 18;18(16):8714. doi: 10.3390/ijerph18168714 (PMC8391803; doi:10.3390/ijerph18168714)
Supplement: Supplementary file 1 [file ijerph-18-08714-s001.zip › ijerph-1291311-supplementary.pdf]

*Quick Environmental Exposure and Sensitivity Inventory (QEESI) Publications by Country

Austria

Weiss, E. M., Singewald, E., Baldus, C., Hofer, E., Marksteiner, J., Nasrouei, S., . . . Holzer, P. (2017). Differences in psychological and somatic symptom cluster score profiles between subjects with Idiopathic environmental intolerance, major depression and schizophrenia. *Psychiatry Research, 249*, 187-194. doi:10.1016/j.psychres.2016.12.057

China

Huang, L.-L., Ikeda, K., Chiang, C.-M., Kagi, N., Hojo, S., & Yanagi, u. (2011). Field Survey on the Relation between IAQ and Occupants' Health in 40 Houses in Southern Taiwan. *Journal of Asian Architecture and Building Engineering, 10*. doi:10.3130/jaabe.10.249

Huang, L.-l., Ikeda, K., Hojo, S., Kagi, N., & Juan, H.-C. (2014). Study of the different Cutoff Point of the QEESI Questionnaire as a Screening Tool for Sick Building Syndrome Diagnosis in Taiwan. *Journal of Asian Architecture and Building Engineering, 13*(2), 507-513. doi:10.3130/jaabe.13.507

Columbia

Storino, V., Muñoz-Ortiz, J., Villabona-Martinez, V., Villamizar-Sanjuán, J. D., Rojas-Carabali, W., & de-la-Torre, A. (2021). An Unusual Case of Multiple Food Allergies Comorbid with Multiple Chemical Sensitivity: A Case Report. *Journal of asthma and allergy*, *14*, 317–323. https://doi.org/10.2147/JAA.S293248

Denmark

Dantoft, T. M., Elberling, J., Brix, S., Szecsi, P. B., Vesterhauge, S., & Skovbjerg, S. (2014). An elevated pro-inflammatory cytokine profile in multiple chemical sensitivity. *Psychoneuroendocrinology, 40*, 140-150. doi:10.1016/j.psyneuen.2013.11.012

Hauge, C. R., Rasmussen, A., Piet, J., Bonde, J. P., Jensen, C., Sumbundu, A., & Skovbjerg, S. (2015). Mindfulness-based cognitive therapy (MBCT) for multiple chemical sensitivity (MCS): Results from a randomized controlled trial with 1 year follow-up. *Journal of Psychosomatic Research, 79*(6), 628-634. doi:10.1016/j.jpsychores.2015.06.010

Skovbjerg, S., Berg, N. D., Elberling, J., & Christensen, K. B. (2012). Evaluation of the Quick Environmental Exposure and Sensitivity Inventory in a Danish Population. *Journal of Environmental and Public Health, 2012*, 304314. doi:10.1155/2012/304314

Tran, M. T. D., Skovbjerg, S., Arendt-Nielsen, L., Bech, P., Lunde, M., & Elberling, J. (2014). Two of three patients with multiple chemical sensitivity had less symptoms and secondary hyperalgesia after transcranially applied pulsed electromagnetic fields. *Scand J Pain, 5*(2), 104-109. doi:10.1016/j.sjpain.2013.11.008

Tran, M. T. D., Skovbjerg, S., Arendt-Nielsen, L., Christensen, K. B., & Elberling, J. (2017). A randomised, placebo-controlled trial of transcranial pulsed electromagnetic fields in patients with multiple chemical sensitivity. *Acta Neuropsychiatrica. Officieel Wetenschappelijk Orgaan van Het IGBP (Interdisciplinair Genootschap voor Biologische Psychiatrie), 29*(5), 267-277. doi:10.1017/neu.2016.51

Finland

Heinonen-Guzejev, M., Koskenvuo, M., Mussalo-Rauhamaa, H., Vuorinen, H., Heikkil‰, K., & Kaprio, J. (2012). Noise sensitivity and multiple chemical sensitivity scales: Properties in a population based epidemiological study. *Noise and Health, 14*(60), 215-223. doi:10.4103/1463-1741.102956

Selinheimo, S., Vuokko, A., Hublin, C., Järnefelt, H., Karvala, K., Sainio, M., . . . Paunio, T. (2019). Health-related quality among life of employees with persistent nonspecific indoor-air-associated health complaints. *Journal of Psychosomatic Research, 122*, 112-120. doi:10.1016/j.jpsychores.2019.03.181

Vuokko, A., Karvala, K., Suojalehto, H., Lindholm, H., Selinheimo, S., Heinonen-Guzejev, M., . . . Sainio, M. (2019). Clinical Characteristics of Disability in Patients with Indoor Air-Related Environmental Intolerance. *Safety and health at work, 10*(3), 362-369. doi:10.1016/j.shaw.2019.06.003

France

Kamoun, H., Romdhane, N. A., Rekik, W., Laadheri, N., Youssef, I., Ben Salah, F., & Gharbi, R. (2011). Multiple chemical sensitivity syndrome. Observation of a cohort of 20 workers. *Archives des Maladies Professionnelles et de l'Environnement*, *72*(1), 73-79. https://doi.org/10.1016/j.admp.2010.08.006

Germany

Bauer, A., Schwarz, E., Martens, U. (2007). Patients with multiple chemical sensitivities: A case for environmental or psychosomatic medicine? Zeitschrift fur Allgemeinmedizin, 83(11):442-446.

Schnakenberg, E., Fabig, K. R., Stanulla, M., Strobl, N., Lustig, M., Fabig, N., & Schloot, W. (2007). A cross-sectional study of self-reported chemical-related sensitivity is associated with gene variants of drug-metabolizing enzymes. *Environmental health : a global access science source*, *6*, 6. https://doi.org/10.1186/1476-069X-6-6

Indonesia

Hildebrandt, S., Kubota, T., Sani, H. A., & Surahman, U. (2019). *Indoor Air Quality and Health in Newly Constructed Apartments in Developing Countries: A Case Study of Surabaya, Indonesia*. *Atmosphere.* 10(4):182

Kubota, T., Sani, H. A., Hildebrandt, S., & Surahman, U. (2020). Indoor air quality and self-reported multiple chemical sensitivity in newly constructed apartments in Indonesia. *Architectural Science Review*. https://doi.org/10.1080/00038628.2020.1779647

Italy

Caccamo, D., Cesareo, E., Mariani, S., Raskovic, D., Ientile, R., Currò, M., . . . De Luca, C. (2013). Xenobiotic sensor- and metabolism-related gene variants in environmental sensitivity-related illnesses: a survey on the Italian population. *Oxidative Medicine and Cellular Longevity, 2013*, 831969. doi:10.1155/2013/831969

De Luca, C., Scordo, M. G., Cesareo, E., Pastore, S., Mariani, S., Maiani, G., . . . Korkina, L. G. (2010). Biological definition of multiple chemical sensitivity from redox state and cytokine profiling and not from polymorphisms of xenobiotic-metabolizing enzymes. *Toxicology and Applied Pharmacology, 248*(3), 285-292. doi:10.1016/j.taap.2010.04.017

De Luca, C., Thai, J. C., Raskovic, D., Cesareo, E., Caccamo, D., Trukhanov, A., & Korkina, L. (2014). Metabolic and genetic screening of electromagnetic hypersensitive subjects as a feasible tool for diagnostics and intervention. *Mediators of Inflammation, 2014*, 924184. doi:10.1155/2014/924184

De Luca, C., Gugliandolo, A., Calabrò, C., Currò, M., Ientile, R., Raskovic, D., . . . Caccamo, D. (2015). Role of Polymorphisms of Inducible Nitric Oxide Synthase and Endothelial Nitric Oxide Synthase in Idiopathic Environmental Intolerances. *Mediators of Inflammation, 2015*, 245308. doi:10.1155/2015/245308

Gugliandolo, A., Gangemi, C., Calabrò, C., Vecchio, M., Di Mauro, D., Renis, M., . . . Caccamo, D. (2016). Assessment of glutathione peroxidase-1 polymorphisms, oxidative stress and DNA damage in sensitivity-related illnesses. *Life Sciences, 145*, 27-33. doi:10.1016/j.lfs.2015.12.028

Micarelli, A., Viziano, A., Bruno, E., Micarelli, E., & Alessandrini, M. (2016). Vestibular impairment in Multiple Chemical Sensitivity: Component analysis findings. *Journal of Vestibular Research, 26*(5-6), 459-468. doi:10.3233/ves-160594

Micarelli, A., Viziano, A., Genovesi, G., Bruno, E., Ottaviani, F., & Alessandrini, M. (2016). Lack of contralateral suppression in transient-evoked otoacoustic emissions in multiple chemical sensitivity: a clinical correlation study. *Noise Health, 18*(82), 143-149. doi:10.4103/1463-1741.181997

Viziano, A., Micarelli, A., & Alessandrini, M. (2017). Noise sensitivity and hyperacusis in patients affected by multiple chemical sensitivity. *International Archives of Occupational and Environmental Health, 90*(2), 189-196. doi:10.1007/s00420-016-1185-8

Japan

Azuma, K., Uchiyama, I., Takano, H., Tanigawa, M., Azuma, M., Bamba, I., & Yoshikawa, T. (2013). Changes in cerebral blood flow during olfactory stimulation in patients with multiple chemical sensitivity: a multi-channel near-infrared spectroscopic study. *PloS One, 8*(11), e80567. doi:10.1371/journal.pone.0080567

Azuma, K., Uchiyama, I., Katoh, T., Ogata, H., Arashidani, K., & Kunugita, N. (2015a). Prevalence and Characteristics of Chemical Intolerance: A Japanese Population-Based Study. *Archives of Environmental & Occupational Health, 70*(6), 341-353. doi:10.1080/19338244.2014.926855

Azuma, K., Uchiyama, I., Tanigawa, M., Bamba, I., Azuma, M., Takano, H., . . . Sakabe, K. (2015b). Assessment of cerebral blood flow in patients with multiple chemical sensitivity using near-infrared spectroscopy--recovery after olfactory stimulation: a case-control study. *Environmental Health and Preventive Medicine, 20*(3), 185-194. doi:10.1007/s12199-015-0448-4

Azuma, K., Uchiyama, I., Tanigawa, M., Bamba, I., Azuma, M., Takano, H., . . . Sakabe, K. (2016). Association of Odor Thresholds and Responses in Cerebral Blood Flow of the Prefrontal Area during Olfactory Stimulation in Patients with Multiple Chemical Sensitivity. *PloS One, 11*(12), e0168006. doi:10.1371/journal.pone.0168006

Azuma, K., Uchiyama, I., & Kunugita, N. (2019). Factors affecting self-reported chemical intolerance: A five-year follow-up study in Japan. *Journal of Psychosomatic Research, 118*, 1-8. doi:10.1016/j.jpsychores.2019.01.001

Cui, X., Lu, X., Hiura, M., Oda, M., Miyazaki, W., & Katoh, T. (2013). Evaluation of genetic polymorphisms in patients with multiple chemical sensitivity. *PloS One, 8*(8), e73708-e73708. doi:10.1371/journal.pone.0073708

Cui, X., Lu, X., Hiura, M., Oda, M., Hisada, A., Miyazaki, W., . . . Katoh, T. (2014). Prevalence and interannual changes in multiple chemical sensitivity in Japanese workers. *Environmental Health and Preventive Medicine, 19*(3), 215-219. doi:10.1007/s12199-014-0378-6

Cui, X., Lu, X., Hisada, A., Fujiwara, Y., & Katoh, T. (2015). The correlation between mental health and multiple chemical sensitivity: a survey study in Japanese workers. *Environmental Health and Preventive Medicine, 20*(2), 123-129. doi:10.1007/s12199-014-0434-2

Fujimori, S., Hiura, M., Yi, C. X., Xi, L., & Katoh, T. (2012). Factors in genetic susceptibility in a chemical sensitive population using QEESI. *Environmental Health and Preventive Medicine, 17*(5), 357-363. doi:10.1007/s12199-011-0260-8

Hasegawa, M., Ohtomo, M., Mizuki, M., & Akiyama, K. (2009). [Diagnosis of multiple chemical sensitivity by chemical compounds exposure tests]. *Arerugi, 58*(2), 112-118.

Hojo, S. (2002). A questionnaire survey of multiple chemical sensitivity in Japan by using QEESI. *Neuro-Ophthalmology Japan, 19*(2), 169-175.

Hojo, S., Kumano, H., Yoshino, H., Kakuta, K., & Ishikawa, S. (2003). Application of Quick Environment Exposure Sensitivity Inventory (QEESI©) for Japanese population: study of reliability and validity of the questionnaire. *Toxicology and Industrial Health, 19*(2-6), 41-49. doi:10.1191/0748233703th180oa

Hojo, S., Yoshino, H., Kumano, H., Kakuta, K., Miyata, M., Sakabe, K., . . . Ishikawa, S. (2005). Use of QEESI© questionnaire for a screening study in Japan. *Toxicology and Industrial Health, 21*(3-4), 113-124. doi:10.1191/0748233705th219oa

Hojo, S., Ishikawa, S., Kumano, H., Miyata, M., & Sakabe, K. (2008). Clinical characteristics of physician-diagnosed patients with multiple chemical sensitivity in Japan. *International Journal of Hygiene and Environmental Health, 211*(5-6), 682-689. doi:10.1016/j.ijheh.2007.09.007

Hojo, S., Sakabe, K., Ishikawa, S., Miyata, M., & Kumano, H. (2009). Evaluation of subjective symptoms of Japanese patients with multiple chemical sensitivity using QEESI©. *Environmental Health and Preventive Medicine, 14*, 267-275.

Hojo, S., Mizukoshi, A., Azuma, K., Okumura, J., Ishikawa, S., Miyata, M., . . . Sakabe, K. (2018). Survey on changes in subjective symptoms, onset/trigger factors, allergic diseases, and chemical exposures in the past decade of Japanese patients with multiple chemical sensitivity. *International Journal of Hygiene and Environmental Health, 221*(8), 1085-1096. doi:10.1016/j.ijheh.2018.08.001

Hojo, S., Mizukoshi, A., Azuma, K., Okumura, J., Mizuki, M., & Miyata, M. (2019). New criteria for multiple chemical sensitivity based on the Quick Environmental Exposure and Sensitivity Inventory developed in response to rapid changes in ongoing chemical exposures among Japanese. *PloS One, 14*(4), e0215144-e0215144. doi:10.1371/journal.pone.0215144

Ishibashi, M., Tonori, H., Miki, T., Miyajima, E., Kudo, Y., Tsunoda, M., Sakabe, K., & Aizawa, Y. (2007). Classification of patients complaining of sick house syndrome and/or multiple chemical sensitivity. *Tohoku Journal of Experimental Medicine*, *211*(3), 223-233. https://doi.org/10.1620/tjem.211.223

Lu, X., Hisada, A., Anai, A., Nakashita, C., Masuda, S., Fujiwara, Y., . . . Katoh, T. (2020). Study of the Correlation Between Multiple Chemical Sensitivity and Personality Using the Quick Environmental Exposure Sensitivity Inventory Questionnaire and the Temperament and Character Inventory. *Journal of Occupational and Environmental Medicine, 62*(7), e348-e354. doi:10.1097/jom.0000000000001899

Manabe, R., Kunugita, N., Katoh, T., Kuroda, Y., Akiyama, Y., Yamano, Y., . . . Arashidani, K. (2008). [Questionnaire survey of workers in specific buildings regarding multiple chemical sensitivity]. *Nihon Eiseigaku Zasshi. Japanese Journal of Hygiene, 63*(4), 717-723. doi:10.1265/jjh.63.717

Mizuki, M. (2004) Effect of environmental factors in patients with multiple chemical sensitivity. *IRYO-Japanese Journal of National Medical Services, 58*(7), 399-407.

Mizuki, M. (2004) Intractable factors in patients with chemical sensitivity. *IRYO-Japanese Journal of National Medical Services, 69*(3), 117-126.

Mizukoshi, A., Kumagai, K., Yamamoto, N., Noguchi, M., Yoshiuchi, K., Kumano, H., . . . Yanagisawa, Y. (2015). In-situ Real-Time Monitoring of Volatile Organic Compound Exposure and Heart Rate Variability for Patients with Multiple Chemical Sensitivity. *International Journal of Environmental Research and Public Health, 12*(10), 12446-12465. doi:10.3390/ijerph121012446

Nakaoka, H., Suzuki, N., Nakayama, Y., Takaya, K., Todaka, E., and Mori, C. (2018). A preliminary study to investigate the relationships between indoor environment and its effect on physical and mental health. WIT Transactions on Ecology and the Environment, *230*, 567-576.

Ohsawa, M., Takayama, S., Kikuchi, A., Arita, R., Saito, N., Hojo, S., . . . Yaegashi, N. (2020). Two Cases of Multiple Chemical Sensitivity Successfully Treated With Kampo Medicine. *Alternative Therapies in Health and Medicine*.

Suzuki, N., Nakaoka, H., Nakayama, Y., Tsumura, K., Takaguchi, K., Takaya, K., . . . Mori, C. (2020). Association between sum of volatile organic compounds and occurrence of building-related symptoms in humans: A study in real full-scale laboratory houses. *Science of the Total Environment, 750*, 141635. doi:10.1016/j.scitotenv.2020.141635

Watai, K., Fukutomi, Y., Hayashi, H., Kamide, Y., Sekiya, K., & Taniguchi, M. (2018). Epidemiological association between multiple chemical sensitivity and birth by caesarean section: a nationwide case-control study. *Environmental Health, 17*(1), 89. doi:10.1186/s12940-018-0438-2

Yoshino, H., Amano, K., Matsumoto, M., Netsu, K., Ikeda, K., Nozaki, A., . . . Ishikawa, S. (2004). Long-Termed Field Survey of Indoor Air Quality and Health Hazards in Sick House. *Journal of Asian Architecture and Building Engineering, 3*(2), 297-303. doi:10.3130/jaabe.3.297

Saudi Arabia

Khalil, A. I., Almutairi, M. S., & Ahmed, M. E. (2020). Assessing risk factors of Autism Spectrum Disorders (ASD) and Attention Deficit Hyperactivity Disorder (ADHD) among Saudi Mothers: A retrospective study [Article]. *Clinical Schizophrenia and Related Psychoses*, *14*(1), Article 092320. https://doi.org/10.3371/CSRP.IASM.092320

South Korea

Heo, Y., Kim, S. H., Lee, S. K., & Kim, H. A. (2017). Factors Contributing to the Self-Reported Prevalence of Multiple Chemical Sensitivity in Public Facility Workers and the General Population of Korea. *J uoeh, 39*(4), 249-258. doi:10.7888/juoeh.39.249

Jeon, B. H. L., S.H.; Kim H.A. (2012). A Validation of the Korean Version of QEESI(c) (The Quick Environmental Exposure and Sensitivity Inventory). *Korean Journal of Occupational and Environmental Medicine., 24*(1), 96-114.

Jeong, I., Kim, I., Park, H. J., Roh, J., Park, J. W., & Lee, J. H. (2014). Allergic diseases and multiple chemical sensitivity in Korean adults. *Allergy, Asthma & Immunology Research, 6*(5), 409-414. doi:10.4168/aair.2014.6.5.409

Yun, M. J., Kang, D. M., Lee, K. H., Kim, Y. K., & Kim, J. E. (2013). Multiple chemical sensitivity caused by exposure to ignition coal fumes: a case report. *Ann Occup Environ Med, 25*(1), 32. doi:10.1186/2052-4374-25-32

Spain

Aguilar-Aguilar, E., Marcos-Pasero, H., de la Iglesia, R., Espinosa-Salinas, I., Ramírez de Molina, A., Reglero, G., & Loria-Kohen, V. (2018). Characteristics and determinants of dietary intake and physical activity in a group of patients with multiple chemical sensitivity. *Endocrinol Diabetes Nutr, 65*(10), 564-570. doi:10.1016/j.endinu.2018.07.009

Alobid, I., Nogué, S., Izquierdo-Dominguez, A., Centellas, S., Bernal-Sprekelsen, M., & Mullol, J. (2014). Multiple chemical sensitivity worsens quality of life and cognitive and sensorial features of sense of smell. *European Archives of Oto-Rhino-Laryngology, 271*(12), 3203-3208. doi:10.1007/s00405-014-3015-5

Fernandez-Solà, J. (2005). Chronic fatigue syndrome and fibromyalgia in patients affected with multiple chemical sensitivity. Med Clin (Barc); 124: 451-453.

García-Sierra, R., & Álvarez-Moleiro, M. (2014). Evaluation of suffering in individuals with multiple chemical sensitivity. *Clínica y Salud, 25*(2), 95-103. doi:https://doi.org/10.1016/j.clysa.2014.06.006

Lago Blanco, E., Puiguriguer Ferrando, J., Rodríguez Enríquez, M., Agüero Gento, L., Salvà Coll, J., & Pizà Portell, M. R. (2016). Multiple chemical sensitivity: Clinical evaluation of the severity and psychopathological profile. *Medicina Clínica*, *146*(3), 108-111. https://doi.org/10.1016/j.medcli.2015.09.016

Mena, G., Sequera, V. G., Nogué-Xarau, S., Ríos, J., Bertran, M. J., & Trilla, A. (2013). [Translation and cross-cultural adaptation of the Quick Environmental Exposure and Sensitivity Inventory for use in the Spanish population]. *Medicina Clínica, 140*(7), 302-304. doi:10.1016/j.medcli.2012.10.024

Nogué, S., Fernández-Solá, J., Rovira, E., Montori, E., Fernández-Huerta, J. M., & Munné, P. (2007). [Multiple chemical sensitivity: study of 52 cases]. *Medicina Clínica, 129*(3), 96-98; quiz 99. doi:10.1157/13107370

Paredes Rizo, M. (2018). Multiple Chemical Sensitivity: analysis of a case registered in a reference Hospital. *Medicine and Occupational Safety*, *64* (251), 217-240.

Pérez-Crespo, J., Lobato-Cañón, R., & Solanes-Puchol, Á. (2018). Multiple Chemical Sensitivity in Chemical Laboratory Workers. *Safety and health at work, 9*(4), 473-478. doi:10.1016/j.shaw.2018.03.001

Sweden

Andersson, M. J., Andersson, L., Bende, M., Millqvist, E., & Nordin, S. (2009). The idiopathic environmental intolerance symptom inventory: development, evaluation, and application. *Journal of Occupational and Environmental Medicine, 51*(7), 838-847. doi:10.1097/JOM.0b013e3181a7f021

Nordin, S., & Andersson, L. (2010). Evaluation of a Swedish version of the Quick Environmental Exposure and Sensitivity Inventory. *International Archives of Occupational and Environmental Health, 83*(1), 95-104. doi:10.1007/s00420-009-0427-4

United States

Gould Peek, G., & Wallace Lyon, M. (2014). Housing and Health Relationship: Multiple Chemical Sensitivity (MCS). *Housing and Society, 41*(1), 31-52. doi:10.1080/08882746.2014.11430620

Heilbrun, L. P., Palmer, R. F., Jaen, C. R., Svoboda, M. D., Perkins, J., & Miller, C. S. (2015). Maternal Chemical and Drug Intolerances: Potential Risk Factors for Autism and Attention Deficit Hyperactivity Disorder (ADHD). *Journal of the American Board of Family Medicine, 28*(4), 461-470. doi:10.3122/jabfm.2015.04.140192

Katerndahl, D. A., Bell, I. R., Palmer, R. F., & Miller, C. S. (2012). Chemical intolerance in primary care settings: prevalence, comorbidity, and outcomes. *Annals of Family Medicine, 10*(4), 357-365. doi:10.1370/afm.1346

Miller, C. S., & Prihoda, T. J. (1999b). The Environmental Exposure and Sensitivity Inventory (EESI): a standardized approach for measuring chemical intolerances for research and clinical applications. *Toxicology and Industrial Health, 15*(3-4), 370-385. doi:10.1177/074823379901500311

Miller, C. S., & Prihoda, T. J. (1999b). A controlled comparison of symptoms and chemical intolerances reported by Gulf War veterans, implant recipients and persons with multiple chemical sensitivity. *Toxicology and Industrial Health, 15*(3-4), 386-397. doi:10.1177/074823379901500312

Palmer, R. F., Jaén, C. R., Perales, R. B., Rincon, R., Forster, J. N., & Miller, C. S. (2020). Three questions for identifying chemically intolerant individuals in clinical and epidemiological populations: The Brief Environmental Exposure and Sensitivity Inventory (BREESI). *PloS One, 15*(9), e0238296. doi:10.1371/journal.pone.0238296

Uruguay

De Ben, S., Sponton, F. Chaves, E., Medina, F., Tomasina, F. (2014.) Multiple chemical sensitivity: a challenge for occupational health. Rev Med Urug, 30: 123-127.
